# Characterization of the complete chloroplast genome of *Betula pendula* purple rain (betulaceae)

**DOI:** 10.1080/23802359.2023.2176182

**Published:** 2023-02-20

**Authors:** Meiqi Zhang, Yuan Gao, Xiaoyue Su, Weili Liu, Yanli Guo, Jing Jiang, Wei Ma

**Affiliations:** a Forestry College, Northeast Forestry University, Harbin, China; b Chemical Engineering and Resource utilization College, Northeast Forestry University, Harbin, China; c The First Affiliated Hospital of Heilongjiang University of Traditional Chinese Medicine, Harbin, China; d Experiment and Training Center, Heilongjiang University of Chinese Medicine, Harbin, China

**Keywords:** *Betula pendula* purple rain, chloroplast genome, phylogeny, Betulaceae

## Abstract

*Betula pendula* purple rain is a variety of *Betula pendula* that is native to Europe and has important ornamental and economic value. In this study, we sequenced the complete chloroplast genome of *B. pendula* purple rain. This genome had a typical quadripartite structure with 160,552 bases, including a large single copy (LSC) region of 89,433 bases, a small single copy (SCC) region of 19,007 bases and two inverted repeat (IR) regions of 26,056 bases. The GC content of the chloroplast genome was 36% and contained 124 genes, including 79 protein-coding genes, 8 rRNA genes and 37 tRNA genes. The maximum likelihood phylogenetic analysis of reported chloroplast genomes showed that *B. pendula* purple rain was most closely related to *Betula occidentalis* and *Betula platyphylla*.

## Introduction

*B. pendula* purple rain belongs to the family Betulaceae, which occurs from northern Xinjiang to the Altai Mountains, Europe and America (Li et al. 2009). *B. pendula* purple rain is a deciduous tree, up to 25 meters high, that grows in river beach, valley, foot of wet zone or sunny stone hillside areas located 500–2000 meters above sea level. Its leaves are purple, its trunk is grey–white, and its ornamental value is very high (Lin 2014). Chloroplasts are a kind of plastid that mainly exist in some cells of mesophylls and young stems of higher plants. Chloroplasts are also sites of photosynthesis in green plants, where pigments and proteins are synthesized (Xing and Liu 2008). The chloroplast genome of angiosperms consists of four major fragments: the small single copy region (SSC), the large single copy region (LSC), and two inverted repeat (IR) regions. Chloroplasts have the advantages of a relatively conserved structure, moderate base mutation rate and simple sequencing, and they have been widely used in the phylogenetic studies of various plant groups (Greiner et al. 2019). In this paper, for the first time, we report the complete chloroplast genome of *B. pendula* purple rain, which provides more detailed and complete data for the study of chloroplasts in Betulaceae.

## Materials

**Figure 1.**
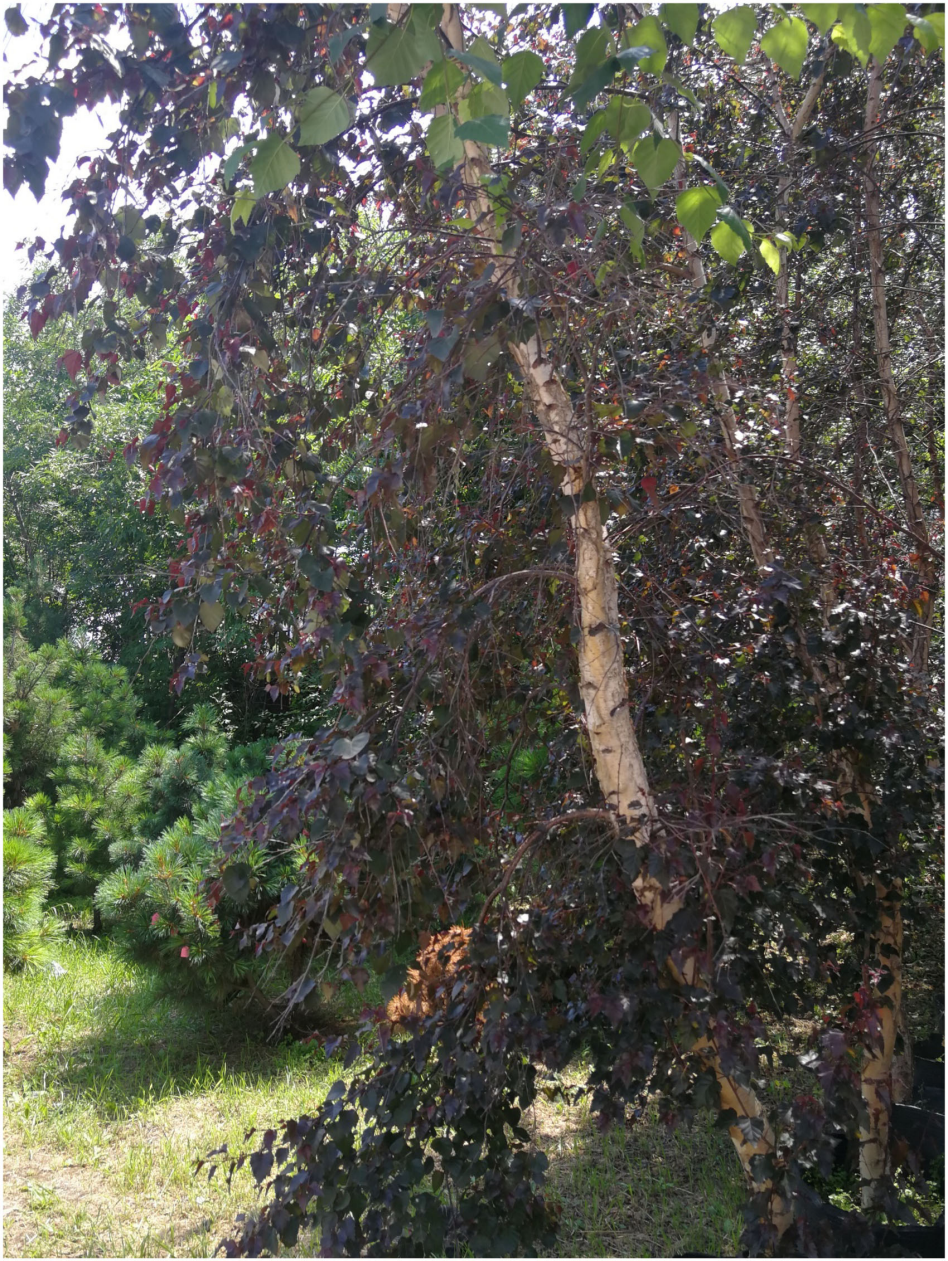
Photo taken at the Forest Genetics and Breeding Base of Northeast Forestry University on July 30, 2022. Eight-year-old *B. pendula* purple rain.

The fresh leaves of *B. pendula* purple rain were collected from eight-year-old plants (N: 45°72′05900′, E: 126°63′4623″) from the Forest Genetics and Breeding Base of Northeast Forestry University on July 30, 2022 (Figure 1). Professor Jiang Jing of Northeast Forestry University identified as *B. pendula* purple rain. The specimens were stored at Heilongjiang University of Chinese Medicine (http://yxy.hljucm.net/, Dr. Zhang, qq77123454829@126.com) (registration number: HRB20220730001).

## Methods

Total genomic DNA of *B. pendula* purple rain was extracted by the CTAB method and stored at Heilongjiang University of Chinese Medicine.Qualified DNA was used to construct a 150 bp paired-end library, which was sequenced by the Illumina NovaSeq^6000^ high-throughput sequencing platform. SOAPnuke software was used to identify and remove low-quality read fragments and missing reads. SPAdes software was used for assembly, and CPGAVAS2 software was used to annotate the complete chloroplast genome sequence of *B. pendula* purple rain (Bankevich et al. 2012; Shi et al. 2019). The chloroplast genome has been submitted to the National Genomics Data Center (NGDC:https://ngdc.cncb.ac.cn/) with the entry number (GSA:CRA008742) (CNCB-NGDC Members and Partners 2021).

## Results

The chloroplast genome has a quadripartite structure with 160,552 bases, including a large single copy (LSC) region of 89,433 bases, a small single copy (SCC) region of 19,007 bases and two inverted repeat (IR) regions of 26,056 bases. The GC content of the chloroplast genome is 36% and contains 124 genes, including 79 protein-coding genes, 8 rRNA genes and 37 tRNA genes. Of these genes, *ycf3* and *clpP* have two introns, whereas *trnK-UUU*, *trnG-GCC*, *atpF*, *rpoC1*, *trnL-UAA*, *trnV-UAC*, *petB*, *petD*, *rpl16*, *rpl2*, *ndhB*, *trnI-GAU*, *trnA-UGC* and *ndhA* have one intron.

**Figure 2.**
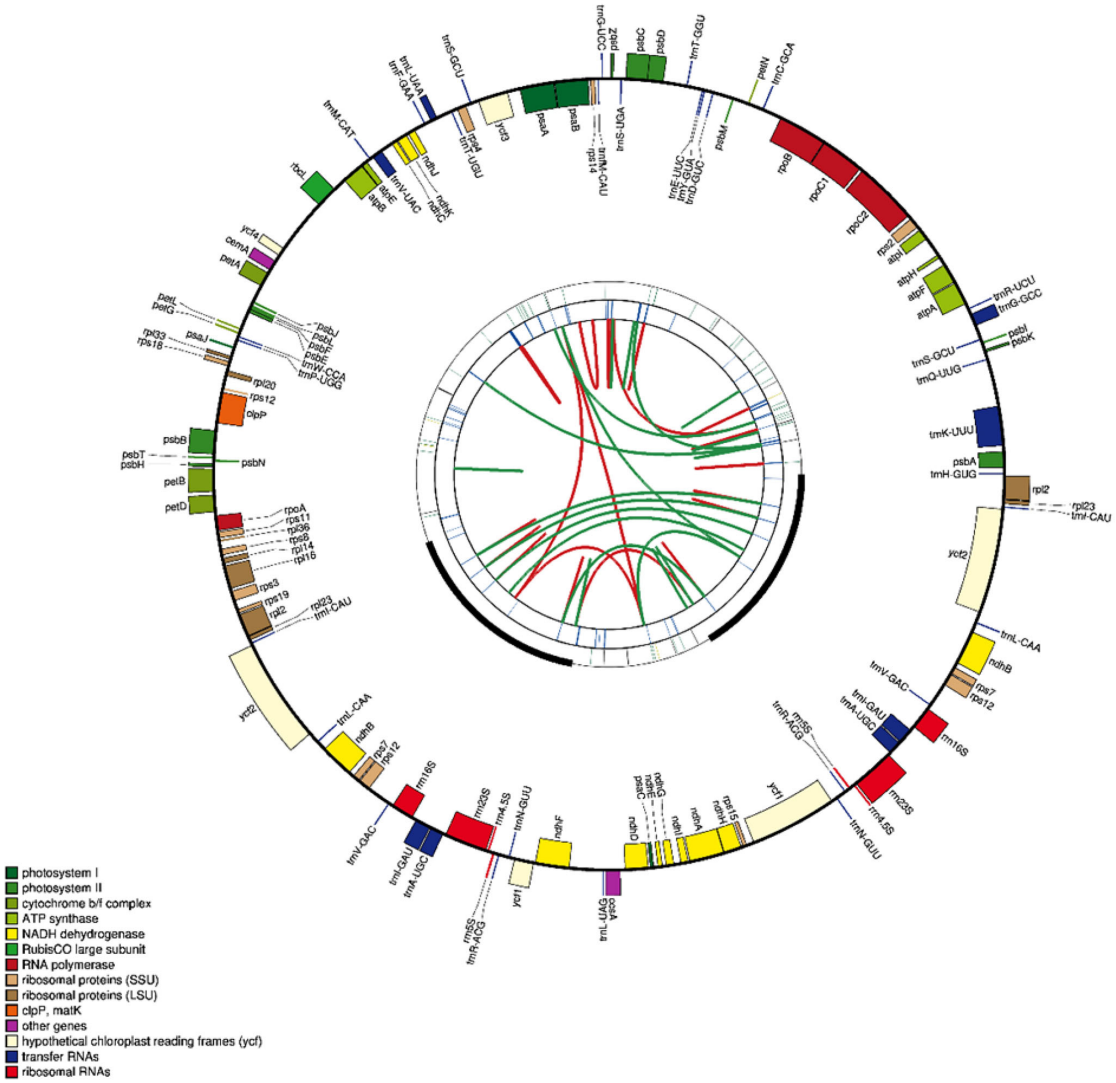
The map contains four rings. From the center going outward, the first circle shows the forward and reverse repeats connected with red and green arcs respectively. The next circle shows the tandem repeats marked with short bars. The third circle shows the microsatellite sequences identified using MISA. The fourth circle is drawn using drawgenemap and shows the gene structure on the plastome. The genes were colored based on their functional categories.

**Figure 3.**
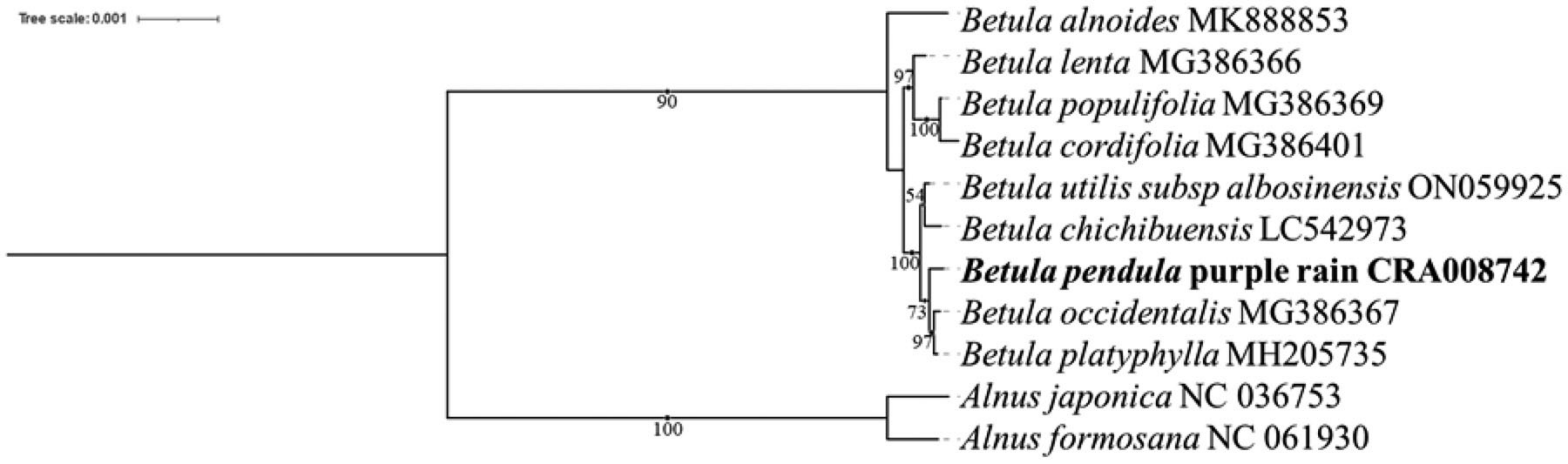
Phylogenetic tree reconstruction of 11 samples using maximum likelihood based on complete chloroplast genome. Note: The sequences of *Betula pendula Purple Rain* was from NGDC.;The remaining sequence data were from NCBI; represents the species in this study.

A comparative chloroplast genome analysis of *B. pendula* purple rain and eight other species previously reported Betulaceae chloroplast genomes (Figure 2) showed that the chloroplast genome ranged in length from 160,263 to 161,123 bp, with a GC content of 36% to 36.4%. Together, these chloroplast genomes contain 72 common genes (Wang et al. 2019; Yoshida et al. 2020). These results suggest that the chloroplast genome of Betulaceae is relatively stable in length, structure and GC content and that the chloroplast genome is relatively conserved. To elucidate the phylogenetic relationship of *B. pendula* purple rain, the complete chloroplast genome sequences of 8 related Betula species were downloaded from NCBI, and the chloroplast genome sequences of 2 Alnus plants were selected as outgroups. Phylosuite software was used to extract common genes, MAFFT software (version: 7.313) was used to match multiple sequences of common genes, IQTREE software (version: v.1.6.8) was used to select the best fitting model (TVM + F + I), and the chloroplast genome evolutionary tree was obtained (Bootstraps: 1000). The chloroplast genome map of *B. pendula* purple rain. (Figure 3).

## Discussion and conclusion

In this study, we first report the complete chloroplast genome of *B. pendula* purple rain characterized by purple leaves. The maximum likelihood phylogenetic analysis of the reported chloroplast genomes showed that *B. pendula* purple rain was most closely related to *B. occidentalis* and *B. platyphylla. B. pendula* purple rain, *B. occidentalis* and *B. platyphylla* was sister to a monophyletic clade of *B. utilis subsp albosinensis* and *B. chichibuensis* at the interspecific level (BS = 100%). The chloroplast genome data of this plant provide more detailed and complete information for the study of evolutionary relationships within Betulaceae, which lays the foundation for plant identification and evolutionary genetic analysis.

## Data Availability

Data availability statement The genome sequence data that support the findings of this study are openly available in the National Genomics Data Center of NGDC at https://ngdc.cncb.ac.cn/ under the accession no. CRA008742. The associated BioProject, GSA, and Bio-Sample numbers are PRJCA012943, CRA008742, and SAMC976969, respectively.
